# Replacement of the Dominant ST191 Clone by ST369 Among Carbapenem-Resistant *Acinetobacter baumannii* Bloodstream Isolates at a Tertiary Care Hospital in South Korea

**DOI:** 10.3389/fmicb.2022.949060

**Published:** 2022-07-14

**Authors:** Seong Eun Kim, Su-Mi Choi, Yohan Yu, Sung Un Shin, Tae Hoon Oh, Seung-Ji Kang, Kyung-Hwa Park, Jong Hee Shin, Uh Jin Kim, Sook In Jung

**Affiliations:** ^1^Department of Infectious Diseases, Chonnam National University Medical School, Gwangju, South Korea; ^2^Department of Laboratory Medicine, Chonnam National University Medical School, Gwangju, South Korea

**Keywords:** Carbapenem-resistant *Acinetobacter baumannii*, multilocus sequence typing (MLST), ST369, replacement, competition, virulence

## Abstract

The clonal dissemination of carbapenem-resistant *Acinetobacter baumannii* (CRAB) bacteremia is a serious clinical problem worldwide. However, the factors related to the emergence and replacement of predominant CRAB clones in nosocomial settings are unclear. By multilocus sequence typing (MLST), we evaluated the genetic relatedness of CRAB bloodstream isolates at a tertiary care hospital over a 3.5-year period and investigated the clinical and microbiologic characteristics of the predominant sequence types (STs). One hundred and seventy-nine CRAB bloodstream isolates were collected from June 2016 to December 2019, and their MLSTs according to Oxford scheme and clinical data were obtained. The predominant STs were assessed for *in vitro* growth, competitive growth, and virulence in a mouse model of intraperitoneal infection. Two dominant clones—ST369 (*n* = 98) and ST191 (*n* = 48)—belonging to international clone 2 (IC2) were recovered from patients admitted to intensive care units (ICUs) or wards. ST191 predominated (61%, 27/43) from June 2016 to July 2017, whereas ST369 (72%, 98/136), which was first isolated from a patient admitted to the emergency room, replaced ST191 (15%, 21/136) after August 2017. In a multivariate analysis, leukopenia (OR = 3.62, 95% CI 1.04–12.6, *p* = 0.04) and ST191 or 369 (OR = 5.32, 95% CI 1.25–22.65, *p* = 0.02) were independent risk factors for 7-day mortality. Compared with non-ST369, ST369 was associated with a shorter time to bacteremia from ICU admission (7 vs. 11 days, *p* = 0.01), pneumonia as an origin of bacteremia (67 vs. 52%, *p* = 0.04), leukopenia (28 vs. 11%, *p* < 0.01), and a lower 7-day survival rate (41 vs. 70%, *p* < 0.01). *In vitro*, ST 369 isolates had significantly higher growth rates and enhanced competitive growth compared to ST191. Finally, ST369 had greater virulence and a higher mortality rate than other STs in a mouse infection model. We report almost-complete replacement of the predominant ST191 clone by ST369 within an 8-month period at our hospital. ST369 had a high incidence density rate of CRAB bacteremia, a short time to bacteremia after ICU admission, and a high early mortality rate, which may be in part explained by its faster competitive growth rate and higher virulence than ST191.

## Introduction

*Acinetobacter baumannii* is an important cause of nosocomial infections, especially in the intensive care unit (ICU; Munoz-Price et al., 2013). The rate of carbapenem-resistant *A. baumannii* (CRAB) is unprecedentedly high, which, combined with clonal transmission, has led to severe clonal outbreaks with high mortality rates in ICU settings (Siempos et al., 2010). CRAB outbreaks have been traced to common-source contamination and to cross-infection by the hands of healthcare workers (Villegas and Hartstein, 2003; Maragakis et al., 2004). CRAB outbreaks often show monoclonality (Lolans et al., 2006) and multilocus sequence typing (MLST) is the reference approach for their epidemiological investigation (Dijkshoorn et al., 1996, 2007; Zarrilli et al., 2013). Two MLST schemes are available for *A. baumannii*. The Oxford scheme is more discriminative among closely related isolates, while the Pasteur scheme is more useful to study the population biology and epidemiological studies of *A. baumannii* and related species (Gaiarsa et al., 2019). One of the most successful clonal lineages, *A. baumannii* IC2, corresponds to clonal complex 92 (CC92) according to MLST using the Oxford scheme (Bartual et al., 2005). Certain sequence types (ST) of *A. baumannii* IC2 not only have regional predominance but also higher mortality rates than STs (Zhou et al., 2018; Yoon et al., 2019). In an epidemiologic study of CRAB in the United States, IC2 (ST2 by the Pasteur Institute scheme) was identified most frequently (Adams-Haduch et al., 2011). In South Korea, MLST of CRAB bloodstream infection (BSI) isolates at a tertiary hospital from 2009 to 2015 showed that ST191 (IC2) accounted for 60% (Kim et al., 2020) and predominated throughout the study. In a multicenter Korean study from 2016 to 2017, ST191 accounted for 40% of *A. baumannii* BSI isolates and had a high 30-day mortality rate (Yoon et al., 2019).

From June 2016 to December 2019, a change in the time to acquire CRAB bacteremia in ICU patients and their mortality was noted at a tertiary hospital. In fatal CRAB bacteremia cases after August 2017, death occurred shortly after detection of bacteremia. We evaluated the MLST-based genetic relatedness of CRAB isolates at a tertiary care hospital over a 3.5-year period. We analyzed 7- and 30-day mortality according to STs and risk factors. In addition, we conducted an *in vitro* growth analysis, competitive growth assay, and an *in vivo* animal study to evaluate the microbiologic characteristics of CRAB isolates, particularly the predominant clones.

## Materials and Methods

### Bacterial Isolates

Carbapenem-resistant *A. baumannii* bloodstream isolates from patients with CRAB between June 2016 and December 2019 at Chonnam National University Hospital (a 1,000-bed tertiary care hospital with eight ICUs in Gwangju, Korea) were stored at −80°C. Species identification was performed initially by matrix-assisted laser desorption ionization-time of flight mass spectrometry-based VITEK MS (bioMérieux, France) and VITEK 2 (bioMérieux) and was confirmed by using MLST-based identification as previously described (Gaiarsa et al., 2019). Carbapenem resistance was initially determined using the VITEK 2 AST Card (bioMérieux) and confirmed by broth microdilution test (Clinical and Laboratory Standards Institute, 2020). Duplicate CRAB isolates from the same patient were excluded.

### Antimicrobial Susceptibility Testing

Antimicrobial susceptibility was tested by the broth microdilution method for 11 anti-*Acinetobacter* drugs of eight classes following the Clinical Laboratory Standards Institute (CLSI) guidelines (Clinical and Laboratory Standards Institute, 2020). Susceptibility categories were interpreted according to the CLSI criteria (Clinical and Laboratory Standards Institute, 2020) for all antibiotics except for tigecycline. Due to the lack of established CLSI breakpoints for tigecycline at this time, Food and Drug administration (FDA) breakpoints issued for Enterobacteriaceae (susceptible ≤2 mg/L and non-susceptible ≥4 mg/L) were applied for interpretation of results (Marchaim et al., 2014).

### Patients and Clinical Data

We collected the following clinical and laboratory information: age, sex, pre-existing illnesses, presence of intensive care unit (ICU) stay before BSI within 2 months, time to BSI after ICU admission or hospitalization, presence of central venous catheter (CVC) within 48 h of BSI, early and late mortality, and appropriate antibiotics. The Charlson comorbidity index score was used to quantify the severity of pre-existing illness (Charlson et al., 1987).

### Definitions

Carbapenem-resistant *A. baumannii* was defined as *A. baumannii* resistant to both imipenem and meropenem by the broth microdilution test based on CLSI breakpoints (Clinical and Laboratory Standards Institute, 2020). CRAB BSI was defined as one or more CRAB positive blood culture. Pneumonia was diagnosed if both of the following criteria were met: (1) progressive pulmonary infiltrate on imaging; (2) clinical finding of respiratory tract infection sign or symptom exists (cough, fever, difficult breathing, increased respiratory rate, or respiratory secretions). Ventilator associated pneumonia (VAP) was defined as pneumonia that develops after more than 48 h of mechanical ventilation. Central line-associated bloodstream infection (CLABSI) was defined as a primary BSI in a patient with a central line within 48 h before development of the BSI, and BSI was not related to an infection at another site according to the Centers for Disease Control definition (Horan et al., 2008). Appropriate antibiotics were defined as in previous studies (Hsieh et al., 2016; Lee et al., 2017): (1) antimicrobial agents were administered as recommended in the Sanford Guide (Antimicrobial Therapy, 2019); (2) CRAB was susceptible *in vitro* to the antimicrobial agent based on the CLSI breakpoints (Clinical and Laboratory Standards Institute, 2020). The minimal inhibitory concentration (MIC) of colistin was defined as ≤2 μg/ml using the CLSI susceptibility breakpoint. Colistin-based therapy was defined as colistin monotherapy or combination therapy without tigecycline. Tigecycline-based therapy was defined tigecycline monotherapy or combination therapy without colistin (Liang et al., 2018). Time to appropriate antibiotics was defined as the period between initial blood culture from which CRAB was isolated and administration of the first appropriate antimicrobials. Clinical outcomes were death within 7 and 30 days. Disease severity was evaluated by sequential organ failure (SOFA) score (Vincent et al., 1996) on the first day of BSI.

### Multilocus Sequence Typing

Strain typing was conducted by multilocus sequence typing (MLST) following the Oxford scheme (Bartual et al., 2005). The housekeeping genes *gltA*, *gyrB*, *gdhB*, *recA*, *cpn60*, *gpi*, and *rpoD* were sequenced (Supplementary Table S1). The isolates were assigned to STs using the tools on PubMLST database.^1^ The population structure of STs of the *A. baumannii* isolates was evaluated using the goeBURST.^2^ The default definition (sharing six of seven alleles) was used to identify international clones.

### *In vitro* Growth

The *in vitro* growth rate was assessed for *A. baumannii* STs and the reference strain ATCC19606. ST191 and ST369 isolates were diluted to 1 × 10^6^ CFU/ml after exponential growth in Luria-Bertani (LB) broth and incubated at 37°C with constant shaking at 180 rpm. At 12, 24, 36, and 48 h, a 10-fold serial dilution was spread on LB agar, and CFUs were enumerated after 24 h of incubation at 37°C. For each strain, three independent experiments were carried out at each time point.

### *In vitro* Competitive Growth Assay

*In vitro* competitive growth of ST369 and ST191 strains was assessed based on the competition index and percent recovery. Competitive growth of *A. baumannii* ST369 was assessed in a 1:1 mixture with *A. baumannii* ST191 strain by a PCR method (Liu et al., 2016). Briefly, *A. baumannii* ST369 and ST191 were separately cultured overnight in LB broth at 37°C. The bacteria were diluted 1:100, and equal CFU numbers of ST369 and ST191 were cocultured at 37°C for 24 h. CFUs were counted, and 20 randomly selected colonies were subjected to PCR to detect *gpi*106 (ST369) or *gpi*94 (ST191). The competition index was determined as follows: competition index = [CFU of isolates positive for *gpi*106 (ST 369) after 24 h cocultured ÷ CFU of isolates positive for *gpi*94 (ST191) after 24 h cocultured] ÷ [CFU of ST369 isolates inoculated ÷ CFU of ST191 isolates inoculated]. Three independent experiments were performed.

Percent recovery was evaluated by modifying a method described previously (Hafza et al., 2018). We used the gentamicin-susceptible ST369 *A. baumannii* strain, and randomly selected five gentamicin-resistant ST369 and five gentamicin-resistant ST191 *A. baumannii* isolates to create five combinations. Each set of bacterial isolates was separately cultured overnight in LB broth at 37°C and diluted 1:100. Gentamicin-resistant ST369 and ST191 were pooled with an equal number of the gentamicin-susceptible ST369 isolate, cocultured in LB broth at 37°C for 24 h, and serially diluted 1:10 in PBS. Next, 20 μl of each dilution were spread on LB agar and selective LB agar containing gentamicin (32 μg/ml). The CFU of the original suspensions was calculated by counting viable colonies on agar plates after overnight incubation at 37°C. For each combination, we determined the numbers of CFUs of (A) gentamicin-resistant ST369 and gentamicin-susceptible ST369 on LB agar, (B) gentamicin-resistant ST369 and gentamicin-susceptible ST369 on LB agar containing gentamicin, (C) gentamicin-resistant ST191 and gentamicin-susceptible ST369 on LB agar, and (D) gentamicin-resistant ST191 and gentamicin-susceptible ST369 on LB agar containing gentamicin. Percent recovery of ST369 was calculated as B ÷ A × 100 and of ST191 as D ÷ C × 100. The experiment was repeated three times for each set of combinations.

### *In vivo* Animal Study

Female, specific pathogen-free, 8-week-old BALB/c female mice of average weight 20 g (Samtako, Osan Republic of Korea) and *A. baumannii* ATCC19606 (American Type Culture Collection, Manassas, VA, United States), ST369, ST191, and ST784 were used in the study. For intraperitoneal (ip) inoculation in mice, freshly cultured inocula were prepared from frozen stocks of *A. baumannii* as described previously (Harris et al., 2017). Inocula were enumerated by plating 10-fold serial dilutions on LB agar. Mice were observed for mortality over 6 days.

### Statistical Analysis

The Kolmogorov–Smirnov goodness-of-fit test was used to determine the normality of the data distribution. Categorical variables are expressed as percentages. Continuous variables are expressed as means ± SD if normally distributed and as medians and interquartile range (Q1, Q3) if non-normally distributed. In univariate analyses, Pearson’s chi-squared or Fisher’s exact test was used for comparisons of categorical variables and Student’s *t*-test (normal distribution), Mann–Whitney test, or Kruskal–Wallis test (non-normal distribution) for continuous variables. Time-series count data corresponding to the incidence density of CRAB bacteremia were analyzed using a Poisson regression model. Growth was evaluated by paired *t*-test as described previously (Pournaras et al., 2014). A Kaplan–Meier survival analysis and log-rank test were performed to compare mortality according to ST. Values of *p* ≤ 0.05 were deemed to indicate statistical significance. Statistical analysis was performed in SPSS Statistics (version 25, IBM Corp., Armonk, NY, United States) and Prism (ver. 8.0; GraphPad Software, La Jolla, CA, United States) software.

## Results

### Multilocus Sequence Typing

**Figure 1:**
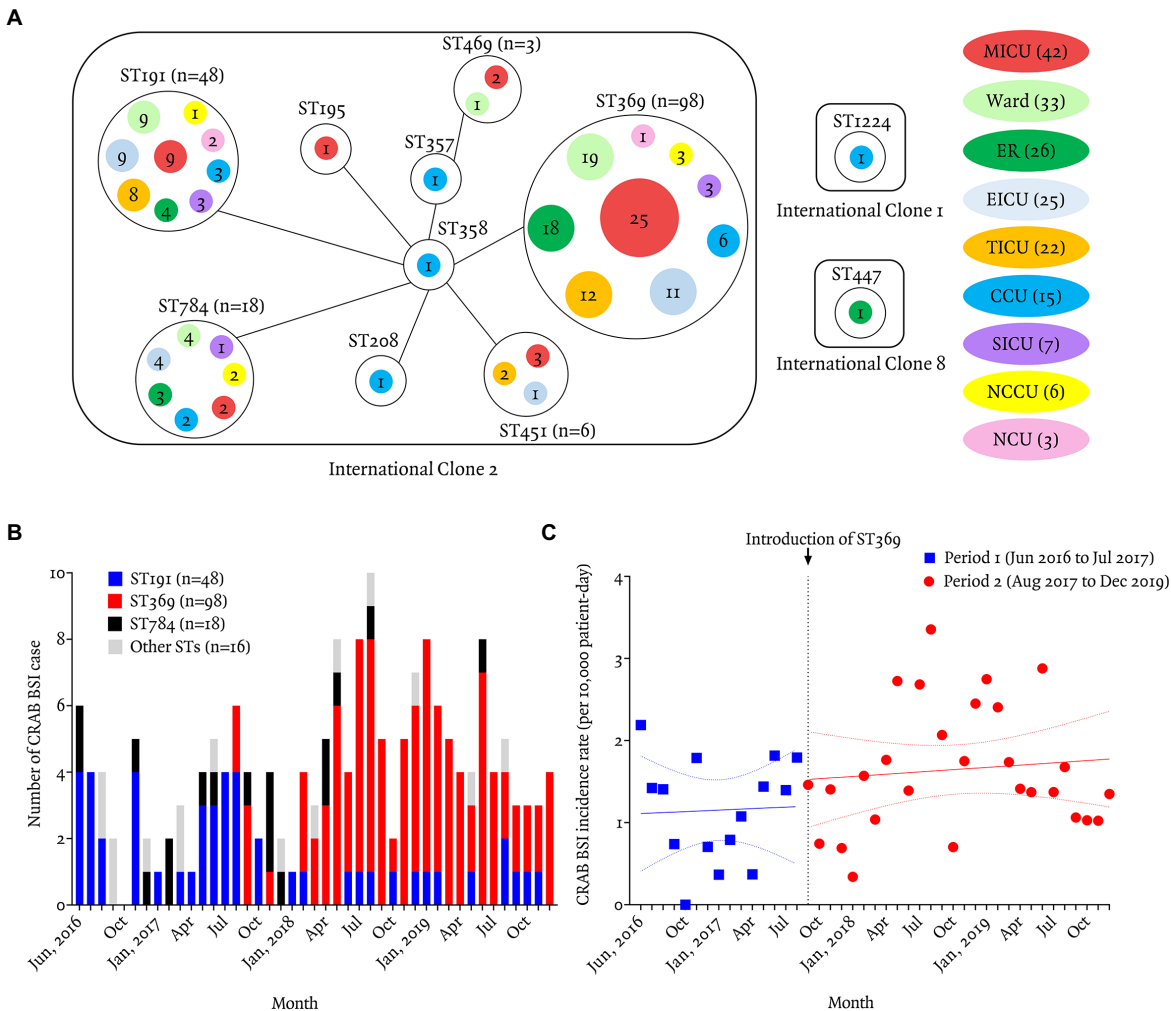
Geographic distribution and incidence of carbapenem-resistant *Acinetobacter baumannii* bacteremia according to sequence typing. **(A)** Relationships between the 12 STs and patients’ locations at the time of bacteremia. Of the 12 STs, nine (ST369, ST191, ST784, ST451, ST469, ST195, ST357, ST358, and ST208) were classified as IC2, and ST447, ST1224, and ST674 as IC8, IC1, and unclassified IC, respectively. Most isolates (177/180, 98%) belonged to IC2. ST369 and ST191 were recovered from the same ICUs (MICU, EICU, TICU, CCU, SICU, NCCU, or NCU), ER. or wards, but ST369 isolates showed a greater propensity for clonal spread than ST191 isolates. Solid line represents one genetic mismatch among seven housekeeping genes. Colored circles are the locations of patients at the time of bacteremia. Numbers in colored circles are numbers of CRAB BSI cases. MICU, medical intensive care unit; ER, emergency room; TICU, trauma intensive care unit; EICU, emergency intensive care unit; CCU, coronary critical care unit; SICU, surgical intensive care unit; NCCU, neurologic critical care unit; NCU, neurosurgical care unit. **(B)** Monthly number of carbapenem-resistant *Acinetobacter baumannii* (CRAB) bloodstream infection (BSI) patients from June 2016 to December 2019. ST369 was introduced in August 2017 and predominated thereafter. ST191 (27/44, 61%) predominated from June 2016 to July 2017. ST369 (98/136, 72%) replaced ST191 (21/136, 15%) after August 2017. **(C)** Monthly incidence of CRAB BSI during period 1 (June 2016 to July 2017) was 1.18 cases per 10,000 patient-days (95% CI 0.81–1.54) and period 2 (August 2017 to December 2019) was 1.65 cases per 10,000 patient-days (95% CI 1.36–1.95). The incidence rate ratio was 1.40 (95% CI 1.38–1.43, *p* < 0.01; Poisson regression analysis).

A total of 179 bloodstream isolates were identified as CRAB by both MLST-based identification and resistance to carbapenem during the study period. MLST identified 11 distinct STs. ST369 (55%, 98/179) was the most abundant ST, followed by ST191 (27%, 48/179), 784 (10%, 18/179), and ST451 (3%, 6/179). Using the default definition (sharing six of seven alleles), nine STs (99%, 177/179, exceptions were ST447 and 1,224) were assigned to IC2. Among 179 cases, most patients were in seven ICUs (67%, 120/179) at the onset of bacteremia, followed by a general ward (18%, 33/179) and the emergency room (15%, 26/179). ST369 was most abundant in the medical ICU (26%, 25/98), followed by ward (19%, 19/98), emergency room (18%, 18/98), and trauma ICU (12%, 12/98; Figure 1A).

The first ST369 isolates were introduced in August 2017 by a patient transferred from another hospital to the emergency room. ST191 was the most abundant (63%, 27/43) during period from June 2016 to July 2017 (period 1), whereas ST369 (72%, 98/136) replaced ST191 (15%, 21/136) during period from August 2017 to December 2019 (period 2; Figure 1B). The monthly incidence density rate of CRAB bacteremia increased significantly from 1.15 case per 10,000 patient-days (PD; 95% CI, 0.80–1.51) in period 1–1.65 cases per 10,000 PD (95% CI, 1.36–1.95) in period 2 (incidence rate ratio 1.43; 95% CI, 1.29–1.70; *p* < 0.01 by Poisson regression analysis; Figure 1C).

### Antimicrobial Resistance

Most isolates were susceptible to tigecycline (98%, 175/179), minocycline (98%, 176/179), and colistin (99%, 177/179), and no isolate were susceptible to cephalosporin or quinolone (Supplementary Table S2). ST369 was more susceptible to amikacin and gentamicin than non-ST369 (amikacin, 41 vs. 9%, *p* < 0.01; gentamicin, 39 vs. 6%, *p* < 0.01; Supplementary Table S2).

### Clinical Characteristics and Outcomes of Patients With CRAB Bacteremia According to ST

The sex ratio was 118:61 (male: female) and the median age was 72 (62, 80) years. All CRAB bacteremia was healthcare associated and most patients (93%, 166/179) had one or more comorbidities. About half of patients were on mechanical ventilation (59%, 105/179) or had a CVC (49%, 87/179) prior to bacteremia. Most patients (78%, 140/179) had a history of ICU admission within 2 months prior to bacteremia. The most frequent source of bacteremia was pneumonia (60%, 108/179), followed by CLABSI (13%, 23/179) and skin and soft tissue infection (12%, 22/179). Adequate antibiotics were administered within 48 h in 62 patients (35%; Table 1).

**Table 1:** Clinical characteristics, treatment, and outcome of patient with carbapenem-resistant *Acinetobacter baumannii* bacteremia according to sequence type.

	All (*n* = 179)	ST369 (*n* = 98)	ST191 (*n* = 48)	ST784 (*n* = 18)	Other STs (*n* = 15)	*p* Value (ST369 vs. non-ST369)	*p* Value (ST369 vs. ST191)
Age, year, and median (IQR)	72 (62, 80)	74 (63, 80)	70 (57, 80)	76 (64, 81)	71 (55, 82)	0.28	0.12
Male sex, no. (%)	118 (66)	68 (69)	27 (56)	12 (67)	11 (69)	0.28	0.12
Underlying disease, no. (%)							
Hypertension	88 (49)	46 (47)	25 (52)	10 (56)	7 (47)	0.51	0.56
Diabetes mellitus	64 (36)	31 (32)	20 (42)	7 (39)	6 (38)	0.21	0.23
Chronic lung disease	26 (15)	16 (16)	4 (8)	1 (6)	5 (33)	0.45	0.19
Cerebrovascular disease	35 (20)	20 (20)	11 (23)	2 (11)	2 (13)	0.75	0.73
Chronic kidney disease	26 (15)	12 (12)	8 (17)	4 (22)	2 (13)	0.34	0.47
Charlson comorbidity index, median (IQR)	4 (3, 6)	4 (3.8, 6)	4 (2, 6)	5 (4, 6.3)	5 (3, 7)	0.87	0.34
Clinical status prior to bacteremia, no. (%)							
ICU admission	140 (78)	75 (77)	36 (75)	16 (89)	13 (81)	0.55	0.84
Time to occur bacteremia after ICU admission, days, median (IQR)	9 (5, 16)	7 (4, 14)	9 (6.3, 16.5)	9.5 (5.3, 27.8)	13 (11.5, 16.5)	0.01	0.09
CVC	87 (49)	44 (45)	25 (52)	10 (56)	8 (53)	0.28	0.41
Mechanical ventilator	105 (59)	60 (61)	29 (60)	10 (56)	6 (40)	0.44	0.93
Surgery within 30 days	40 (22)	19 (19)	14 (29)	2 (11)	5 (31)	0.30	0.18
Previous use of antibiotics	166 (93)	90 (92)	45 (94)	17 (94)	14 (93)	0.61	0.68
Sulbactam	1 (1)	0 (0)	1 (2)	0 (0)	0 (0)	0.27	0.15
Tazobactam	93 (52)	44 (45)	29 (60)	11 (61)	9 (60)	0.04	0.08
Carbapenem	58 (32)	27 (28)	17 (35)	10 (56)	4 (25)	0.13	0.33
Quinolone	66 (37)	42 (43)	14 (29)	7 (39)	3 (19)	0.07	0.11
Cephalosporin	83 (46)	48 (49)	23 (48)	8 (44)	4 (25)	0.49	0.90
Aminoglycoside	7 (4)	4 (4)	2 (4)	0 (0)	1 (6)	0.92	0.96
Origin of bacteremia^b^, no. (%)							
Pneumonia	108 (60)	66 (67)	26 (54)	8 (44)	8 (53)	0.04	0.12
Ventilator associated	73 (68)	45 (69)	20 (77)	6 (75)	2(25)	0.78	0.46
CLBSI	23 (13)	8 (8)	10 (21)	4 (22)	1 (7)	0.04	0.03
Skin and soft tissue infection	22 (12)	11 (11)	7 (15)	2 (11)	2 (13)	0.63	0.56
Urinary tract infection	3 (2)	1 (1)	0 (0)	1 (6)	1 (6)	0.45	0.48
Intra-abdominal infection	5 (3)	3 (3)	0 (0)	1 (6)	1 (6)	0.81	0.22
Other infection^c^	7 (4)	5 (5)	1 (2)	0 (0)	1 (6)	0.37	0.39
Unidentified	11 (6)	4 (4)	4 (8)	2 (11)	1 (6)	0.21	0.29
SOFA score^a^, mean ± SD	9.8 ± 4.5	10.1 ± 4.5	10.2 ± 4.6	8.4 ± 4.3	7.1 ± 3.8	0.26	0.88
Laboratory findings							
Leukopenia (< 4,000/mm3), no. (%)	35 (20)	27 (28)	6 (13)	1 (6)	1 (7)	<0.01	0.04
Creatinine, mg/dL, median (IQR)	1.01 (0.6, 1.8)	1.0 (0.6, 1.7)	1.1 (0.7, 2.0)	1.0 (0.9, 2.4)	0.7 (0.5, 1.1)	0.82	0.75
C-reactive protein, mg/dL, media (IQR)	10.3 (5.2, 16.7)	10.5 (4.9, 17.2)	11.8 (6.2, 17.8)	10.2 (5.7, 13.5)	6.5 (2.0, 10.9)	0.62	0.65
Treatment, no. (%)							
Adequate antibiotics within 48 h	62 (35)	33 (34)	14 (29)	7 (39)	8 (50)	0.64	0.77
Colistin-based therapy	55 (89)	32 (97)	13 (93)	4 (57)	6 (75)	0.02	0.17
Tigecycline-based therapy	7 (11)	1 (3)	1 (7)	3 (43)	2 (25)	0.03	0.56
Outcome^d^, no. (%)	*n* = 172	*n* = 96	*n* = 45	*n* = 17	*n* = 14		
7-day survival	92 (53)	39 (41)	26 (58)	15 (88)	12 (86)	<0.01	<0.01
30-day survival	63 (37)	30 (31)	17 (38)	8 (47)	8 (57)	<0.01	0.13

*Categorical variables were compared using chi-square test*.

*Continuous variables were compared by using one-way ANOVA or Kruskal–Wallis test according to the normal or non-normal distribution*.

a *Sequential organ failure (SOFA) scores were available in 42 patients in ST191, 92 in ST369, 15 in ST784, and 12 in other STs*.

b *One patient might have more than one disease*.

c *Includes thrombophlebitis, post-operative meningitis, and septic arthritis*.

d *Seven patients were follow-up loss. Outcome analysis was performed in 172 patients (45 patients in ST191, 96 in ST369, 17 in ST784, and 14 in other STs)*.

*ST, sequence type; ICU, intensive care unit; CVC, central venous catheter; SOFA, sequential organ failure score; CLBSI, central line-associated blood stream infection; and NA, non-applicable*

For ST369, the median time to bacteremia from ICU admission was shorter than non-ST369 [7 (4, 14) days vs. 11 (7, 17) days, OR 1.03 95% CI 1.00–1.06, *p* = 0.07 by binary logistic regression]. As an origin of bacteremia, the frequency of pneumonia was higher in ST369 than non-ST369 [66/98 (67%) vs. 42/81 (52%), OR 1.92 95% CI 1.04–3.51, *p* = 0.04]. The incidence of leukopenia at the time of bacteremia was higher in patients with ST369 than non-ST369 [27/98 (28%) vs. 8/81 (11%), OR 3.47 95% CI 1.48–8.15, *p* < 0.01; Table 1]. Compared to ST191, ST369 was associated with lower frequency of CLBSI [8/98 (8%) vs. 10/48 (21%), OR 2.96 95% CI 1.09–8.08, *p* = 0.03] and higher incidence of leukopenia [27/98 (28%) vs. 6/58 (13%), OR 2.66 95% CI 1.02–6.98, *p* = 0.04; Table 1].

**Figure 2:**
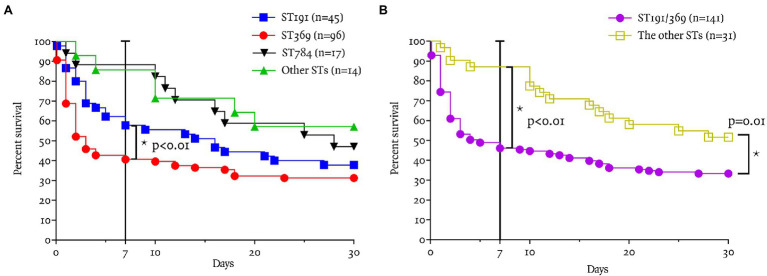
Seven and 30-day survival rates according to ST. **(A)** The 7-day survival rate in patients with ST369 was significantly lower (41%, 39/96) than with the other STs (*p* < 0.01; log-rank test). The 7-day survival rate of ST369 BSI was significantly lower than that of ST191 (41 vs. 58%, *p* = 0.02), other STs (41 vs. 86%, *p* < 0.01), and ST784 (41 vs. 88%, *p* < 0.01). **(B)** The 7-day survival rate in patients with ST191 or ST369 was significantly lower (46%, 65/141) than that of the other STs (87%, 27/31; *p* < 0.01; log-rank test). The 30-day survival rate in patients with ST191 or ST369 was significantly lower (33%, 47/141) than that of the other STs (52%, 16/31; *p* = 0.01; log-rank test).

The 7-day survival rate of CRAB bacteremia was 53% (92/172). The 7-day survival rate in patients with ST369 was significantly lower (41%, 39/96) than for ST191, ST784, and other STs (41% vs. 58, 88, and 86%, respectively; *p* < 0.01; log-rank test, each; Figure 2A). In 7-day mortality cases, the median time to death after detection of bacteremia was shorter in patients with ST369 than ST191 [1 (1, 2) vs. 3 (1, 4) days, *p* = 0.01; Mann–Whitney test]. The 30-day survival rate of ST369 was 31% (30/96), similar to ST191 and ST784 (31% vs. 38 and 47%, respectively; *p* = 0.13 and 0.07; log-rank test; Figure 2A). However, in 30-day mortality cases, the median time to death after bacteremia caused by ST369 was 2 (1, 3) days, which was shorter than for the other STs (*p* < 0.01, Kruskal–Wallis test; all *p* < 0.05 in *post hoc* analysis). The median time to death after bacteremia onset was 4 (2, 14), 12 (6, 21), and 10 (4, 19) days for ST191, ST784, and other STs, respectively.

The 7- and 30-day survival rates of CRAB bacteremia caused by ST369 or ST191 were lower than those for the other STs (46 vs. 87%, *p* < 0.01; 33 vs. 52%, *p* = 0.01; Figure 2B).

### Risk Factors for 7- and 30-Day Mortality in CRAB Bacteremia

We next evaluated risk factors for 7- and 30-day mortality in CRAB bacteremia (Supplementary Table S3). In 7-day fatal group, patients had more underlying diabetes mellitus (43 vs. 28%, *p* = 0.04) and higher Charlson comorbidity index score [5 (4, 6) vs. 4 (2, 6), *p* = 0.049]. Mechanical ventilator was more frequently used in fatal group (69 vs. 50%, *p* = 0.01). SOFA score and serum creatinine was higher in fatal group, compared to nonfatal group [12.4 ± 4.0 vs. 7.6 ± 3.6, *p* < 0.01 and 1.3 (0.8, 2.0) vs. 0.9 (0.6, 1.5), *p* < 0.01]. In addition, leukopenia (36 vs. 7%, *p* < 0.01) and pneumonia (71 vs. 50%, *p* < 0.01) were more frequent in fatal group in univariate analysis. In fatal group, the proportion of CRAB bacteremia caused by ST191 or ST369 was higher than that of nonfatal group (95 vs. 71%, *p* < 0.01). In a multivariate analysis, Charlson comorbidity index score (OR = 1.37, 95% CI 1.06–1.76, *p* = 0.01) and SOFA score (OR = 1.47, 95% CI 1.27–1.70, *p* < 0.01), leukopenia (OR = 3.62, 95% CI 1.04–12.6, *p* = 0.04), and CRAB bacteremia caused by ST191 or 369 (OR = 5.32, 95% CI 1.25–22.65, *p* = 0.02) were independent risk factors for 7-day mortality.

In risk factor analysis for 30-day mortality, fatal group had older age [74 (66, 81) vs. 71 (57, 76) years, *p* = 0.01], more underlying hypertension and diabetes (56 vs. 38%, *p* = 0.02, 40 vs. 25%, *p* = 0.047) and higher Charlson comorbidity index score [5 (4, 6) vs. 4 (2, 6), *p* < 0.01]. The time interval from ICU admission to occur bacteremia was shorter in fatal group than nonfatal group [8 (4, 14) vs. 11 (6, 18) days, *p* = 0.047]. The proportion of mechanical ventilator and pneumonia was higher in fatal group [66 vs. 46%, *p* = 0.01, 68 vs. 46%, *p* = 0.01]. Fatal group had higher SOFA score and creatinine [11.5 ± 4.2 vs. 6.9 ± 3.3, *p* < 0.01 and 1.2 (0.8, 2.0) vs. 0.7 (0.5, 1.2), *p* < 0.01]. Only an elevated SOFA score was an independent risk factor for 30-day mortality (OR = 1.45, 95% CI 1.24–1.68, *p* < 0.01) in multivariate analysis (Table 2).

**Table 2:** Multivariate logistic regression analysis of prognostic factors for 7-day and 30-day mortality in 172 patients with carbapenem-resistant *Acinetobacter baumannii* bacteremia.

	7-day mortality			30-day mortality		
	Adjusted odds ratio	95% CI	*p* Value		Adjusted odds ratio	95% CI	*p* Value
Age					1.00	0.96–1.05	0.92
Hypertension					1.70	0.66–4.41	0.59
Diabetes mellitus	1.00	0.36–2.76	0.99		1.19	0.43–3.29	0.84
Chronic kidney disease	1.66	0.34–8.13	0.60				
Charlson comorbidity index	1.37	1.06–1.76	0.01^*^		1.27	0.93–1.75	0.14
Mechanical ventilator prior to bacteremia	1.80	0.59–5.53	0.31		1.41	0.51–3.91	0.51
SOFA score	1.47	1.27–1.70	<0.01^*^		1.45	1.24–1.68	<0.01^*^
Previous quinolone use					1.35	0.55–3.30	0.51
Leukopenia (<4,000/mm^3^)	3.62	1.04–12.60	0.04^*^		2.20	0.58–8.30	0.25
Creatinine	1.22	0.83–1.79	0.30		1.08	0.76–1.54	0.66
Pneumonia	1.59	0.59–4.29	0.31		1.63	0.65–4.05	0.29
Adequate antibiotics within 48 h	0.54	0.22–1.34	0.19				
Sequence type 191 or 369	5.32	1.25–22.65	0.02^*^		1.22	0.41–3.61	0.73

* *The asterisk indicates the p value less than 0.05*.

### *In vitro* Growth and Competition Assays

**Figure 3:**
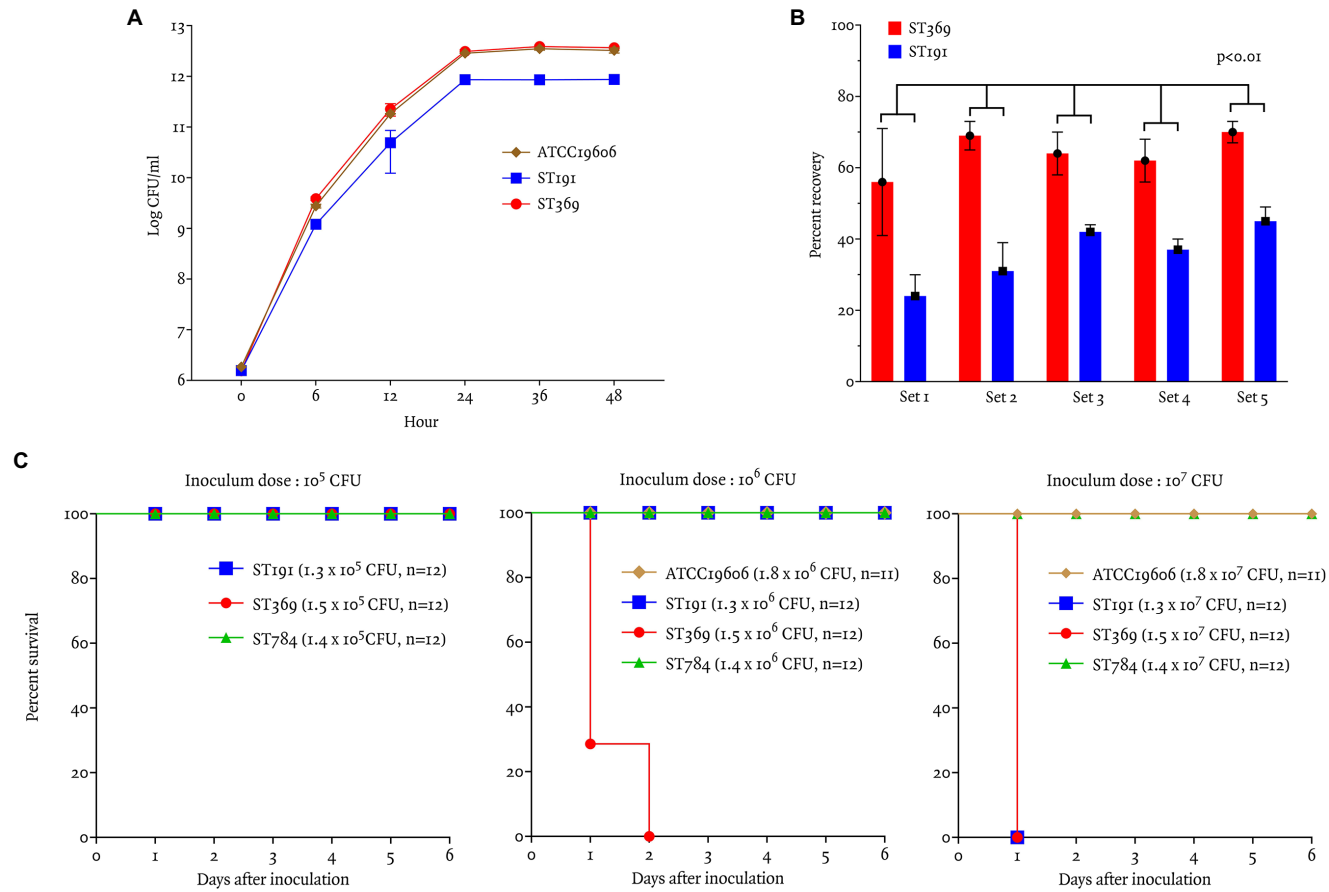
Virulence traits and bacterial fitness of ST191 and ST369 CRAB bloodstream isolates as determined by *in vitro* growth and competition assays and *in vivo* in a mouse model of intraperitoneal infection. **(A)** *In vitro* growth curves of ATCC19606, ST191, and ST369. There were significant differences in the ST191 and ST369 counts after 6 h of incubation at 37°C (*p* = 0.01 at 12 h, *p* < 0.01 at all other time points; Student’s *t*-test). Growth curve of ATCC19606 and ST369 showed similar result and the difference of colony counts were not significant except 6 h of incubation (*p* = 0.02 at 6 h, *p* > 0.05 at all other time points; Student’s *t*-test). **(B)** Competitive growth of 10 gentamicin-resistant (five ST369 and five ST191) *Acinetobacter baumannii* isolates after 24 h of coculture with gentamicin-susceptible *A. baumannii* at 35°C. Percent recovery of ST369 was significantly higher than that of ST191 in all five combinations (*p* < 0.01; Mann–Whitney test). **(C)** Survival rates of BALB/c mice following ip inoculation with *A. baumannii* ATCC19606, ST191, ST369, and ST784. Inoculum was 10^5^–10^7^ CFU and mice were monitored for 6 days. All mice survived inoculation of 10^5^ CFU and only mice inoculated with 10^6^ CFU ST369 showed mortality within 48 h. Mice inoculated with 10^7^ CFU ST369 or ST191 died after 24 h, but those inoculated with ATCC19606 or ST784 survived.

The growth curves of ST191, ST369, and ATCC19606 are shown in Figure 3A. ST369 and ST191 showed significant differences in cell counts at all time points except baseline (*p* < 0.05; Student’s *t*-test), indicating that ST369 had a higher growth rate than ST191 (Figure 3A).

*In vitro*, the competition index of ST369 compared with ST191 was 2.15 ± 0.37 at 24 h after inoculation. Percent recovery of ST369 was higher than that of ST191 after 24 h [66% (58, 69) vs. 36% (30, 42), *p* < 0.01; Mann–Whitney test; Figure 3B].

### *In vivo* Animal Study

We compared the survival rates of mice after ip inoculation with ST191, ST369, ST784, and ATCC19606 (Figure 3C). All mice survived ip inoculation of 10^5^ CFU ST191, ST369, or ST784. After inoculation of 10^6^ CFU, only mice in the ST369 group died within 48 h. After inoculation of 10^7^ CFU ST191 or ST369, all mice died within 24 h but all mice inoculated with 10^7^ CFU ST784 or ATCC19606 survived.

## Discussion

*Acinetobacter baumannii* ST is an important determinant of the incidence of nosocomial CRAB bacteremia and of its severity and mortality. We report almost-complete replacement of the predominant ST191 clone as a cause of CRAB bacteremia by newly introduced ST369 within an 8-month period at our hospital; the incidence density rate of CRAB bacteremia increased thereafter. This observation was supported by the higher growth rate and competitive ability of ST369, compared to ST191 strains, as well as the hypervirulence of the former in a mouse model of infection. Our data highlight the need to be aware of the more rapid onset of ST369 CRAB bacteremia after ICU admission and its poor 7-day clinical outcomes, together with their greater potential for clonal spread.

Although increased prevalence of CRAB has been reported in community-onset bacteremia without identifiable healthcare associated risk factors (Porter et al., 2014; Rhodes et al., 2019) CRAB is a common cause of hospital outbreaks of bacteremia associated with patient carriage or persistence on hospital surfaces (Peleg et al., 2008; Nutman et al., 2016). Clonal transmission or replacement of CRAB occurs occasionally in a nosocomial setting of a hospital (Schultz et al., 2016). MLST analysis of 180 CRAB BSI isolates revealed that ST191 predominated during period 1, consistent with previous reports (Yoon et al., 2019; Kim et al., 2020). However, the ST369 strain replaced ST191 as a cause of CRAB bacteremia during period 2. During the study period, the patient population and the antibiotic selection and ICU environmental management strategies were unchanged. ST369 and ST191 isolates were recovered from the same ICUs, ER, or wards, but the monthly incidence density rate increased significantly in period 2, suggesting that ST369 has greater propensity for clonal spread than ST191. In addition, ST369 had a markedly higher growth rate than ST191, which may in part explain the rapid replacement of the latter by the former and the increased incidence density rate of CRAB bacteremia caused by ST369.

Some STs have high mortality rates in clinical settings (Zhou et al., 2018; Yoon et al., 2019). Patients with clinical isolates of ST457 from blood or respiratory tract had increased endemicity and a higher 7-day mortality rate (44%) than other STs (14%) in China (Zhou et al., 2018). In this study, the 30-day mortality rate of CRAB bacteremia caused by ST191 was 62%, similar to a previous multicenter Korean study, which reported differing 30-day mortality rates of CRAB bacteremia according to ST (ST191, 60.3%; ST451, 17.2%; Yoon et al., 2019). There was no significant difference in the 30-day mortality rate of CRAB bacteremia caused by ST191 (62%) and ST369 (69%), and both were higher than other STs (17–59%; Da Silva et al., 2018; Zhou et al., 2018; Yoon et al., 2019).

ST369 strains were among CRAB BSI isolates in a Korean institute (Kim et al., 2020) and among clinical isolates from multiple body sites in a Chinese burn institute (Huang et al., 2016). However, the clinical and microbiological characteristics of clonal ST369 isolates of *A. baumannii* from CRAB bacteremia are unclear. CRAB bacteremia caused by ST369 was associated with a shorter time to bacteremia from ICU admission (7 vs. 11 days), pneumonia as an origin of bacteremia (66 vs. 52%), and leukopenia (28 vs. 11%), compared to non-ST369 isolates. In addition, the 7-day mortality rate of patients with CRAB bacteremia caused by ST369 was significantly higher (59%) than for ST191 (42%) and other STs (30%). *Acinetobacter baumannii* virulence can be assessed by detecting virulence factors, such as the outer membrane protein OmpA, phospholipases, efflux pumps, penicillin-binding proteins, outer membrane vesicles (Mcconnell et al., 2013; Antunes et al., 2014), and genes related to iron or biofilm (Li et al., 2020), or using *in vivo* mouse models (Harris et al., 2013, 2019). In this study, ST369 strains exhibited higher virulence than ST191 or ST784 strains in a mouse model of ip infection. We postulate that the rapid growth of ST369 strains enabled them to replace ST191 strains on environmental surfaces in multiple ICUs or wards where vulnerable patients have respiratory ventilators or catheter lines. Alternatively, ST369 strains may be rapidly converted from colonizers to infectious strain, to cause CRAB bacteremia, which may result in leukopenia or early death. In the present study, leukopenia was more common in CRAB bacteremia caused by ST369 and was identified as a significant risk factor for 7-day mortality, which is consistent with the report (Belok et al., 2021). Leukocytes are the first lines of host–microbe interaction when *A. baumannii* invade the host immune system (Tilley et al., 2014). Further studies are needed on the relationship between rapid growth of ST369, leukopenia, and poor outcome.

This study had several limitations. First, clinical data were collected from medical records retrospectively. Second, virulence mechanisms according to ST were not analyzed. Despite these limitations, our findings suggest that new clonal CRAB strains with poor early outcomes may be introduced from outside hospitals and become endemic clones. Therefore, our hospital enacted heightened infection control measures for patients with CRAB to prevent further spread of ST369 strains, and mandated more thorough cleaning of environmental surfaces, accompanied by environmental sampling to ensure removal of reservoirs.

## Data Availability Statement

All datasets generated for this study are included in the article and available at Harvard dataverse.

## Ethics Statement

The Institutional Review Board of Chonnam National University Hospital approved this study (No. CNUH-2019-120). A waiver of consent was granted given the retrospective nature of the clinical analyses. Animal experiments were conducted in compliance with the guidelines of the Institutional Animal Care and Use Committee (IACUC) of Chonnam National University and the Korean Food and Drug Administration. The study protocol was approved by the IACUC (CNU IACUC-H-2020-21) of Chonnam National University Hwasun Hospital.

## Author Contributions

SK, JS, UK, and SI conceived and designed the study. YY, SS, and TO collected the clinical samples and data. SK and S-MC performed the experiments. SK, YY, SS, TO, and UK analyzed the data. SK, UK, S-JK, and K-HP contributed to data summary. SK, JS, UK, and SI contributed to the writing of the manuscript. All authors contributed to the article and approved the submitted version.

## Funding

This work was supported by grants from the Research Institute of Medical Sciences, Chonnam National University Medical School (2012-CURIMS-DR005). The funders had no role in study design, data collection and analysis, decision to publish, or preparation of the manuscript.

## Conflict of Interest

The authors declare that the research was conducted in the absence of any commercial or financial relationships that could be construed as a potential conflict of interest.

## Publisher’s Note

All claims expressed in this article are solely those of the authors and do not necessarily represent those of their affiliated organizations, or those of the publisher, the editors and the reviewers. Any product that may be evaluated in this article, or claim that may be made by its manufacturer, is not guaranteed or endorsed by the publisher.

## Footnotes

^1^ https://pubmlst.org/organisms/acinetobacter-baumannii/

^2^ http://www.phyloviz.net/goeburst/
