# Supplementary material for: Replacement of the Dominant ST191 Clone by ST369 Among Carbapenem-Resistant Acinetobacter baumannii Bloodstream Isolates at a Tertiary Care Hospital in South Korea
Source: Front Microbiol. 2022 Jul 14;13:949060. doi: 10.3389/fmicb.2022.949060 (PMC9335038; doi:10.3389/fmicb.2022.949060)
Supplement: Supplementary file 1 [file Data_Sheet_1.docx]

Table S1. Details of loci and oligonucleotide primers used in the present study.

| Locus | Gene product | Primer | Sequence (5’ to 3’) |
| --- | --- | --- | --- |
| *rpoB* | RNA polymerase subunit B | Ac1055F | GTGATAARATGGCBGGTCGT |
|  |  | Ac1598R | CGBGCRTGCATYTTGTCRT |
| *gltA* | Citrate synthase | Citrato F1 | AATTTACAGTGGCACATTAGGTCCC |
|  |  | Citrato R12 | GCAGAGATACCAGCAGAGATACACG |
| *gyrB* | DNA gyrase subunit B | APRU F | TGTAAAACGACGGCCAGTGCNGGRTCYTTYTCYTGRCA |
|  |  | M13 [-21] | TGTAAAACGACGGCCAGT |
|  |  | UP1E R | CAGGAAACAGCTATGACCAYGSNGGNGGNAARTTYRA |
|  |  | M13 F | CAGGAAACAGCTATGACC |
| *gdhB* | Glucose dehydrogenase B | GDHB 1F | GCTACTTTTATGCAACAGAGCC |
|  |  | GDH SEC F | ACCACATGCTTTGTTATG |
|  |  | GDHB 775R | GTTGAGTTGGCGTATGTTGTGC |
|  |  | GDH SEC R | GTTGGCGTATGTTGTGC |
| *recA* | Homologous  recombination factor | RA1 | CCTGAATCTTCYGGTAAAAC |
|  |  | RA2 | GTTTCTGGGCTGCCAAACATTAC |
| *cpn60* | 60-kDa chaperonin | CPN 3F2 | ACTGTACTTGCTCAAGC |
|  |  | CPN R2 | TTCAGCGATGATAAGAAGTGG |
| *gpi* | Glucose-6-phosphate isomerase | GPI F1 | AATACCGTGGTGCTACGGG |
|  |  | GPI R1 | AACTTGATTTTCAGGAGC |
| *rpoD* | RNA polymerase sigma factor *rpoD* | 70F RPOD | ACGACTGACCCGGTACGCATGTAYATGMGNGARATCGCNACNCT |
|  |  | 70FS | ACGACTGACCCGGTACGCATGTA |
|  |  | 70R RPOD | ATAGAAATAACCAGACGTAAGTTNGCYTCNACCATYTGYTTYTT |
|  |  | 70RS | ATAGAAATAACCAGACGTAAGTT |

Table S2. Antimicrobial Nonsusceptibility Rate of 179 Carbapenem-Resistant *Acinetobacter baumannii* Bloodstream Isolates according to Sequence Types.

|  | No. (%) of isolates | | | |  | |
| --- | --- | --- | --- | --- | --- | --- |
| Antimicrobial agents | ST369 (n=98) | ST191 (n=48) | ST784 (n=18) | Other STs (n=15) | | *P* value |
| Ampicillin/sulbactam | 98 (100) | 48 (100) | 17 (94) | 14 (93) | | <0.01 |
| Ceftriaxone | 98 (100) | 48 (100) | 18 (100) | 15 (100) | | NA |
| Cefepime | 98 (100) | 48 (100) | 18 (100) | 15 (100) | | NA |
| Imipenem | 98 (100) | 48 (100) | 18 (100) | 15 (100) | | NA |
| Meropenem | 98 (100) | 48 (100) | 18 (100) | 15 (100) | | NA |
| Amikacin | 58 (59) | 41 (85) | 18 (100) | 15 (100) | | <0.01 |
| Gentamicin | 60 (61) | 43 (90) | 18 (100) | 15 (100) | | <0.01 |
| Ciprofloxacin | 98 (100) | 48 (100) | 18 (100) | 15 (100) | | NA |
| Colistin | 0 (0) | 1 (2) | 1 (6) | 0 (0) | | 0.18 |
| Minocycline | 0 (0) | 0 (0) | 0 (0) | 3 (20) | | <0.01 |
| Tigecycline | 1 (1) | 3 (6) | 0 (0) | 0 (0) | | 0.17 |

Abbreviations: ST, sequence type; NA, not applicable

Table S3. Comparison of Demographic, Cinical, Laboratory Findings and Treatment between Fatal and Nonfatal groups at 7-day and 30-day in 172 Carbapenem-Resistant *Acinetobacter baumannii* Bacteremia Patients.

|  | | | |  | 7-day Mortality | | |  | 30-day Mortality | | |  |
| --- | --- | --- | --- | --- | --- | --- | --- | --- | --- | --- | --- | --- |
|  | | | | All  (n=172) | Fatal  (n=80) | Nonfatal (n=92) | *P* value |  | Fatal  (n=109) | Nonfatal (n=63) | *P* value |  |
| Age, year, median (IQR) | | | | 72 (61, 80) | 72 (65, 80) | 72 (58, 79) | 0.51 |  | 74 (66, 81) | 71 (57, 76) | 0.01* |  |
| Male sex, no. (%) | | | | 114 (66) | 54 (68) | 60 (65) | 0.75 |  | 70 (64) | 44 (70) | 0.45 |  |
| Underlying disease, no. (%) | | | |  |  |  |  |  |  |  |  |  |
| Hypertension | | | | 85 (49) | 43 (54) | 42 (46) | 0.29 |  | 61 (56) | 24 (38) | 0.02* |  |
| Diabetes mellitus | | | | 60 (35) | 34 (43) | 26 (28) | 0.04* |  | 44 (40) | 16 (25) | 0.047* |  |
| Chronic lung disease | | | | 34 (20) | 17 (21) | 17 (19) | 0.65 |  | 22 (20) | 12 (19) | 0.86 |  |
| Cerebrovascular disease | | | | 26 (15) | 15 (19) | 11 (12) | 0.22 |  | 19 (17) | 7 (11) | 0.27 |  |
| Chronic kidney disease | | | | 26 (15) | 16 (20) | 10 (11) | 0.10 |  | 20 (18) | 6 (10) | 0.12 |  |
| Charlson comorbidity index | | | | 4.5 (3, 6) | 5 (4, 6) | 4 (2, 6) | 0.049* |  | 5 (4, 6) | 4 (2, 6) | <0.01* |  |
| Clinical status prior to bacteremia, no. (%) | | | |  |  |  |  |  |  |  |  |  |
| ICU admission | | | | 134 (78) | 62 (78) | 72 (78) | 0.91 |  | 84 (77) | 50 (79) | 0.73 |  |
| Time to occur bacteremia after ICU admission, days, median (IQR) | | | | 9 (5, 16) | 7 (4, 14) | 9 (5, 16) | 0.07 |  | 8 (4, 14) | 11 (6, 18) | 0.047* |  |
| CVC | | | | 83 (48) | 36 (45) | 47 (51) | 0.43 |  | 52 (48) | 31 (49) | 0.85 |  |
| Mechanical ventilator | | | | 101 (59) | 55 (69) | 46 (50) | 0.01* |  | 72 (66) | 29 (46) | 0.01* |  |
| Previous antibiotics use | | | | 160 (93) | 73 (91) | 87 (95) | 0.40 |  | 102 (94) | 58 (92) | 0.71 |  |
|  | Sulbactam | | | 1 (1) | 0 (0) | 1 (1) | 0.35 |  | 1(1) | 0 (0) | 0.45 |  |
|  | Tazobactam | | | 89 (52) | 37 (46) | 52 (57) | 0.18 |  | 56 (51) | 33 (52) | 0.90 |  |
|  | Carbapenem | | | 55 (32) | 23 (29) | 32 (35) | 0.40 |  | 38 (35) | 17 (27) | 0.29 |  |
|  | Quinolone | | | 65 (38) | 34 (43) | 31 (34) | 0.24 |  | 47 (43) | 18 (29) | 0.06 |  |
|  | Cephalosporin | | | 81 (47) | 38 (48) | 43 (47) | 0.92 |  | 51 (47) | 30 (48) | 0.92 |  |
| Origin of bacteremia ^b^, no. (%) | | | |  |  |  |  |  |  |  |  |  |
|  | Pneumonia | | | 103 (60) | 57 (71) | 46 (50) | <0.01* |  | 74 (68) | 29 (46) | 0.01* |  |
|  |  | | Ventilator associated | 70 (69) | 41(72) | 29(64) | 0.42 |  | 54 (74) | 16 (25) | 0.07 |  |
|  | CLBSI | | | 22 (13) | 8 (10) | 14 (15) | 0.45 |  | 12 (11) | 10 (16) | 0.36 |  |
|  | Skin and soft tissue infection | | | 21 (12) | 9 (11) | 12 (13) | 0.72 |  | 16 (15) | 5 (8) | 0.19 |  |
|  | Urinary tract infection | | | 3 (2) | 1 (1) | 2 (2) | 0.64 |  | 1 (1) | 2 (3) | 0.28 |  |
|  | Intra-abdominal infection | | | 5 (3) | 0 (0) | 5 (5) | 0.03 |  | 1 (1) | 4 (6) | 0.04 |  |
|  | Other infection ^c^ | | | 7 (4) | 2 (3) | 5 (5) | 0.33 |  | 2 (2) | 5 (8) | 0.05 |  |
|  | Unidentified | | | 11 (6) | 3 (4) | 8 (9) | 0.19 |  | 3 (3) | 8 (13) | 0.01* |  |
| SOFA score ^a^, mean±SD | | | | 9.8±4.5 | 12.4±4.0 | 7.6±3.6 | <0.01* |  | 11.5±4.2 | 6.9±3.3 | <0.01* |  |
| Laboratory findings | | | |  |  |  |  |  |  |  |  |  |
| Leukopenia (< 4000/mm3), no. (%) | | | | 35 (20) | 29 (36) | 6 (7) | <0.01* |  | 30 (28) | 5 (8) | <0.01* | |
| Creatinine, mg/dL, media (IQR) | | | | 1.0 (0.6, 1.8) | 1.3 (0.8, 2.0) | 0.9 (0.6, 1.5) | <0.01* |  | 1.2 (0.8, 2.0) | 0.7 (0.5, 1.2) | <0.01* | |
| C-reactive protein, mg/dL, media (IQR) | | | | 10.2 (5.0, 16.6) | 11.8 (4.9, 18.2) | 10.2 (6.1, 15.5) | 0.49 |  | 11.8 (6.2, 17.4) | 9.5 (5.4, 13.7) | 0.23 | |
| Treatment, no. (%) | | | |  |  |  |  |  |  |  |  |  |
|  | Adequate antibiotics within 48 h | | | 60(35) | 21(26) | 39 (42) | 0.03* |  | 37 (34) | 23 (37) | 0.73 |  |
|  |  | Colistin based therapy | | 53 (88) | 20 (91) | 33 (85) | 0.48 |  | 32 (89) | 21 (91) | 0.57 |  |
|  |  | Tigecycline based therapy | | 7 (12) | 1 (5) | 6 (15) | 0.20 |  | 5 (14) | 2 (9) | 0.57 |  |
| Sequence type 191 or 369, no. (%) | | | | 141 (82) | 76 (95) | 65 (71) | <0.01* |  | 94 (86) | 47 (75) | 0.06 |  |

Categorical variables were compared using chi-square test.

Continuous variables were compared by using one-way Student t-test or Mann-Whitney test according to the normal or non-normal distribution.

a. SOFA scores were available 80 patients in nonfatal and 76 in fatal group.

b. One patient might have more than one disease

c. Includes thrombophlebitis, post-operative meningitis, septic arthritis

Abbreviations: ST, sequence type; ICU, intensive care unit; CVC, central venous catheter; SOFA, sequential organ failure score; CLSBI, central line associated bloodstream infection.

* The asterisk indicates the *p* value less than 0.05
